# Green synthesis of multi-functional carbon dots from medicinal plant leaves for antimicrobial, antioxidant, and bioimaging applications

**DOI:** 10.1038/s41598-023-33652-8

**Published:** 2023-04-19

**Authors:** Gangaraju Gedda, Sri Amruthaa Sankaranarayanan, Chandra Lekha Putta, Krishna Kanthi Gudimella, Aravind Kumar Rengan, Wubshet Mekonnen Girma

**Affiliations:** 1 Department of Chemistry, School of Engineering, Presidency University, Bangalore, Karnataka 560064 India; 2 Department of Biomedical Engineering, Indian Institute of Technology Hyderabad, Sangareddy, Telangana 502285 India; 3 Department of Chemistry, School of Science, GITAM (Deemed to Be University), Rudraram, Telangana 502329 India; 4 Department of Chemistry, College of Natural Science, Wollo University, P.O. Box: 1145, Dessie, Ethiopia

**Keywords:** Materials science, Biomaterials, Bioinspired materials

## Abstract

In this research work, carbon dots (CDs) were synthesized from the renewable leaves of an indigenous medicinal plant by the one-pot sand bath method, *Azadirachta indica*. The synthesized CDs were characterized for its optical properties using UV–Vis, Fluorescence and Fourier transform infrared (FT-IR) spectrophotometry and for structural properties using dynamic light scattering (DLS), X-ray Diffraction (XRD) and high resolution Transmission electron microscopy (HR-TEM). The synthesized CDs exhibited concentration dependent biocompatibility when tested in mouse fibroblast L929 cell line. The EC_50_ values of biomedical studies, free radical scavenging activity (13.87 μgmL^−1^), and total antioxidant capacity (38 μgmL^−1^) proved CDs were exceptionally good. These CDs showed an appreciable zone of inhibition when examined on four bacterial (two gram-positive and gram-negative) and two fungal strains at minimum concentrations. Cellular internalisation studies performed on human breast cancer cells (MCF 7- bioimaging) revealed the applicability of CDs in bioimaging, wherein the inherent fluorescence of CDs were utilised. Thus, the CDs developed are potential as bioimaging, antioxidants and antimicrobial agents.

## Introduction

Carbon dots (CDs) have attained significant attention for a vast range of applications developed in sensors, drug delivery, phototherapy, energy storage systems, photocatalysis, and antimicrobial protecting agents^1^. The comprehensive utilization is owing to inherited properties of CDs namely low toxicity^2^, high biocompatibility^3^, tuneable and multi-color fluorescence^4^, photostability^5^, water-solubility^6^, and chemical inertness^7^. Accordingly, CDs are recognized as a potent substitute to conventional fluorescent semiconductor quantum dots, metal/metal-oxide NPs and other forms of carbon nanomaterials in various applications^8–14^. Till now CDs were synthesized from diverse precursors, but still researches are in quest for suitable precursors to enhance the luminescent nature which is supressed due to passivating agents and toxic chemicals. In this course, natural/renewable resource derived fluorescent CDs were synthesized by diverse bottom-up approaches such as hydrothermal/solvothermal, microwave irradiation, ultrasonic, and thermal decomposition techniques^15^. Further employing green synthesis eases fabrication of CDs by subsiding toxic and carcinogenic solvents. Several natural resources like crab shell, prawn shell, green tea leaves, coffee, garlic, ginger, and bio wastes were used as carbon sources in developing fluorescent CDs to overcome surface passivation^16^. Thus, successful fabrication of CDs is probable via a careful selection of precursors with a notable physical and biological importance and employing green approach. A medicinal plant can be a suitable natural resource to develop CDs. The precursor exhibits favoured properties due to chemical composition and bioactive compounds which further do not require surface passivation. Several natural resources like crab shell, prawn shell, green tea leaves^17^, coffee, garlic, ginger, and biowastes have become prominent in developing fluorescent CDs due to their eco-friendliness, low cost, therapeutic properties and high availability^18–20^ in recent times. Hence a suitable natural resource must be chosen to develop CDs that exhibit to experience the best utilization. Furthermore, heteroatoms such as nitrogen (N) and sulphur (S) are the best raw starting materials for CDs when compared to other carbon sources that require additional heteroatom sources. The use of plant parts as green resources not only meets the pressing need for largescale CD production, but also encourages the development of sustainable applications. Because plant parts contain numerous carbohydrates, proteins, amino acids, and other biomolecules that provide sufficient elements for the surface functionality of CDs, they do not require a separate reactant for doping, surface passivation, or post modification. Despite the fact that there are numerous opportunities in the field of plant part derived CDs, there are still numerous challenges in exploring the tremendous potency of plant part derived CDs. Because large precursor compositions may cause heterogeneity in plant part derived CDs, a focused investigation of separation and purification is required^21^. To advance the field, correlations between physical (e.g., size, shape) and chemical (e.g., N: C, O: C) properties of a CD and the resulting optical properties (e.g., peak excitation/emission wavelength, QY) and performance towards various applications must be established.

Neem (*Azadirachta indica*), a medicinal plant known as neem, nimtree or Indian lilac, belongs to the mahogany family^22^. Meliaceae is widespread in tropical regions and subtropical regions. The tree is native to the Indian Subcontinent and Africa. Especially in India, parts of medicinal plant, namely roots, bark, leaves and fruits, benefited to treat a wide range of diseases. Specifically, the active constituents of neem leaves exhibit antipyretic, anti-inflammatory, antibacterial, anti-gastric, antiulcer, antiarthritic, spermicidal, antifungal, antimalarial, hypoglycaemic, immunomodulatory, diuretic and antitumor properties^23^. Isolation of active constituents is a tedious and time-consuming mechanism, also requires carcinogenic solvents. Thus, green synthesis of CDs from medicinal plant materials can be an alternative solution to utilize medicinally important precursors for utilization without usage of toxic solvents, by following minimal purification protocol^24^.

Herein, we describe the synthesis of a promising CDs from neem (*Azadirachta indica*) leaves, as well as the optical properties, antibacterial, antioxidant, antifungal, and bioimaging applications. The protocol used is a one-pot sand bath assisted green method. The fluorescent CDs obtained demonstrated excellent biocompatibility and low-cytotoxicity when tested on Mouse fibroblast cancerous cells (L929). Overall, using the principles of the green approach, self-passivated CDs with high medicinal value are synthesized to investigate free radical scavenging, total antioxidant, antibacterial, and antifungal activities, as well as bioimaging studies. To our knowledge, this is the first study to investigate the synthesis of CDs from neem, and conjugation of the CDs with folic acid (FA) enabled specific targeting to folate receptor-positive MCF-7 cells, as confirmed by in vitro fluorescence imaging. The entire synthetic procedure is quick, repeatable, and scalable, resulting in the synthesis of CDs with excellent photoluminescence properties and low toxicity, establishing CDs as a promising alternative for many therapeutic agents.

## Materials and methods

### Materials

2,2-Diphenyl-1-picrylhydrazyl (DPPH, 98%), Fluconazole, Ascorbic acid, methanol, Ammonium molybdate, Sodium phosphate, Conc. H_2_SO_4_, 3-(4,5-dimethylthiazol-2-yl)-2,5-diphenyltetrazolium bromide (MTT, 97.5%), ethyl (dimethyl aminopropyl) carbodiimide (EDC, 99%), Folic acid (> 98%), N-hydroxysulfosuccinimide sodium salt (sulfo-NHS, 97%) and Dimethyl sulfoxide (DMSO) were purchased from SRL Chemicals, India. All the cell culture consumables were purchased from HiMedia, India unless and otherwise mentioned. MilliQ water was utilized for all the experiments unless and otherwise mentioned.

### Instrumentation

The optical characteristics of CDs & FACDs were evaluated using a UV–visible Spectrophotometer (UV 1800, Shimadzu, Japan), Fourier-transform infrared (FT-IR) spectrophotometer (Bruker ATR-FTIR, USA) and Fluorescence spectrophotometer (RF 6000, Shimadzu, Japan). The fluorescence of the derived carbon dot samples was visualised using a 365 nm UV lamp (Analytik Jena, USA). The hydrodynamic size and zeta potential was estimated by a particle size analyzer (Nicomp 3000, USA). The size and morphological features were analysed using Transmission Electron Microscopy (JEOL JEM 2100) at 200 kV. Cellular internalisation was analysed using fluorescence microscope (Olympus CKX-53, USA).

### Synthesis of CDs from Neem leaves by Sand bath method

The Neem (*Azadirachta indica*) leaves were collected from a garden, GITAM University, Hyderabad, Telangana., India. The collection of plant material and the performance of experimental research on such plants complied with the national guidelines of India. Leaves were thoroughly rinsed with tap water and distilled water to eliminate dust and dirt. The leaves were sun-dried and pulverised into fine powder using agate mortar and pestle. Typically, 2.0 g powder is added to 50.0 mL of demineralized water (DM water) under stirring for one hour. The supernatant was passed through a Whitman filter no.1 paper to separate large and undissolved particulate. The resulted filtrate was carefully collected in to a round bottomed flask and placed on a sand bath^25^. The reflux reaction was maintained at uniform temperature (180 °C) for 12 h under constant stirring. Formation of CDs was confirmed with the development of reddish-brown coloured solution. Thus, the synthesized CDs were purified further by centrifugation, following that by filtration using a 0.22 µm syringe filter. The final CDs were stored at 4 °C and utilized for characterizations and applications.

### Evaluation of Free radical scavenging activity

DPPH assay, a widely used protocol to estimate the free radical scavenging efficiency was adapted following previously registered procedures^20^. The free radical scavenging activity was calcuated by choosing ascorbic acid as a standard reference.

$$Radical\, scavenging\, activity \left({\%}\right)=\frac{Absorbance\, control-Absorbance\, sample}{Absorbance\, control} \times 100{\%}$$

### Evaluation of Total antioxidant capacity

Total antioxidant capacity was estimated following the phosphomolybdate assay^26^. The reagent preparation and method was obtained from previous literature. Ascorbic acid is used as standard reference. The total antioxidant capacity (TAC) of the samples is calculated as tannic acid equivalent (TAE) using the following formula:

$$Total\, antioxidantcapacity\, \left(TAC\right)=\frac{Absorbance\, control - Absorbance\, sample}{Absorbance\, control} \times 100$$

### Antimicrobial activity

#### Antibacterial activity

Agar well diffusion method^27^ was followed to determine the antibacterial activity of CDs. Initially, the autoclaved petri dishes were uniformly distributed with solidifying agar medium, and microbial inoculum was inoculated onto the solidified agar medium surface. Using an aseptic sterile cork borer or a tip, a well of diameter 6 to 8 mm was punched on the surface of agar medium. The each well was supplemented with CDs in different concentrations and subjected for 24 h’ incubation under suitable conditions to observe bacterial growth. The zone of inhibition was calculated in diameter (mm) at each well carefully. The experiment was conducted in triplicates using ciprofloxacin (positive control) and water (negative control).

#### Antifungal activity

Potato dextrose agar slants^28^ were used to subculture fungal strains to obtain a fresh and pure culture after incubating 24–48 h at ambient temperatures. Using DM water, the concentration of the inoculum suspensions was normalized to 1.0 × 10^6^ cells/mL. The inoculum suspension was streaked in three directions over potato dextrose agar medium with a sterile cotton swab. The test samples with various concentrations were added to each well (8 mm diameter) bored on the agar surface and left undisturbed for 4–7 days at 27 °C. The zone of inhibition was measured using a dial calliper. Antifungal agent fluconazole (FLC) and water were chosen as positive and negative controls respectively to determine the sensitivity of fungi.

### Bioimaging studies

#### Conjugation of Folic acid to CDs

EDC-NHS coupling^29^ was followed to conjugate CDs with folic acid. Briefly, 2 mL of 15 mg/mL CDs solution was mixed with 25 mg of sulfo-NHS and 15 mg of EDC in 0.5 M MES buffer solutions was allowed to stir for 1 h under dark conditions. To the activated CDs solution, 2.5 mg of FA solubilized in 1 mL of MES buffer was slowly added dropwise and the mixture was allowed to stir for 24 h. The unreacted materials were removed from the synthesized reaction mixture by dialyzing for 8 h to obtain the final product. The resulted FACDs were evaluated for UV–Vis absorbance, fluorescence and zeta potential^30^.

#### In vitro studies

Mouse fibroblasts cell lines (L929) and Human breast adenocarcinoma cell lines (MCF-7) were purchased from the National Centre for Cell Sciences (NCCS), Pune, India. The DMEM culture media supported with 10% (v/v) Fatal bovine serum (FBS) and 100 U/mL penicillin/streptomycin was used to culture these cell lines. The cells were cultured under aseptic conditions at 37 °C with 5% CO_2_^31^.

#### Biocompatibility of CDs

L929 cell line was used to test the biocompatibility of the CDs. Briefly, in a 96 well plate1 × 10^4^ cell/wells were seeded and incubated for 24 h prior to the treatment with CDs. Post-incubation, the CDs with different concentrations diluted in a media were loaded to each well. Untreated cells were considered as negative control. The percentage cell viability was calculated using MTT assay after 24 h.

#### Bioimaging of CDs and FACDs in cancer cell lines

The bioimaging properties in MCF-7 cells was examined by utilizing the inherent fluorescence properties of CDs and FACDs. On a sterile coverslip, 1 × 10^4^ cells/well were seeded and incubated for 24 h. To these cells, CDs & FACDs were supplemented and subjected under incubation for 24 h. Control cells were maintained without any further addition. The cells were then fixed using 4% Paraformaldehyde pot PBS wash and observed under UV light and red light with a confocal microscope.

## Results and discussion

### Synthesis and characterization of CDs and FACDs

The water-soluble fluorescence CDs from neem (*Azadirachta indica*) leaves were synthesized using a sand bath technique without the usage of passivating agents. The leaf encompasses major active constituents quercetin – a flavonoid, limonoids comprising nimbin derivatives, flavonoids, proteins, carbohydrates, sitosterol and carotene^32^. These active biomolecules comprise carbon, oxygen, nitrogen as core elements. Bottom-up approach is adapted to transform small biomolecules to water soluble nontoxic CDs. Studies are not performed to understand the mechanism of transformation in detail. But, an understanding from previous literature sources that transformation takes place in three major steps. The process initiates hydrolysis and condensation. Subsequently, self-polymerization/aggregation takes place. Finally, transformation to core and surface passivation occurs due to elevated temperature over a period of time^33,34^.

**Figure 1:**
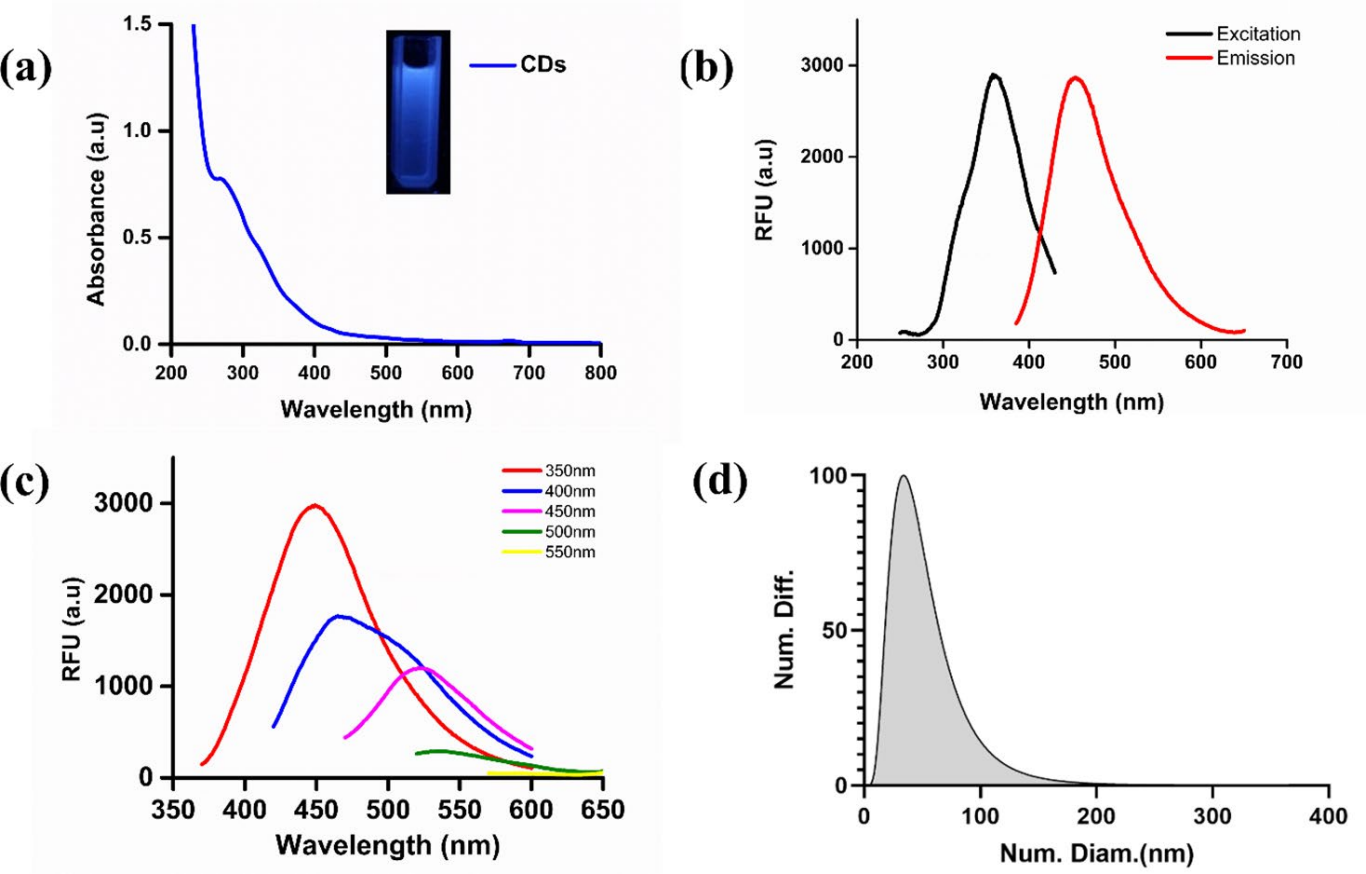
Optical measurements of CDs using UV Visible, fluorescence spectroscopy and DLS technique. (**a**) Ultraviolet absorption spectra of CDs (Inset: Images of CDs under daylight and UV irradiation). (**b**) Excitation and emission spectra of the CDs. (**c**) Fluorescence emission spectra of CDs excitation wavelengths (350 nm to 550 nm range) with increments of 50 nm. (**d**) DLS analysis of CDs.

The CDs were investigated for UV–visible, and fluorescence properties. The CDs exhibited bright blue color fluorescence under exposure to UV light (365 nm) and pale-yellow color in daylight. The obtained UV–Vis absorbance spectra of CDs exhibited peaks at 270, and 350 nm with a tail spread over the visible region (Fig. 1a). The absorption peak at 270 nm was observed due to π-π* transition of the sp^2^ conjugated system in an aromatic ring containing C = C bonds. A peak at 350 nm corresponds to a n-π* transition due to C = O bonds^35^. Excitation and emission CDs spectra in water are shown in Fig. 1b. With an excitation wavelength of 370 nm, the strongest emission peak was at 460 nm. The CDs fluorescence spectra at various excitation wavelengths (350 to 550 nm) range with an increment of 50 nm were recorded, as illustrated in Fig. 1c. The emission wavelengths showed a maximum red shift from 450 to 580 nm, decreasing intensity with the increasing excitation wavelength. The C = O bonds lie as a surface functional group on CDs and give rise to fluorescence by trapping energy on the surface states. The stability of the as prepared CDs was assessed by irradiating with Uv light from 5–180 min as shown in Fig. S1a and there was no difference observed in the absorbance spectra. Furthermore, the CDs were stored for two months and the absorbance spectra were measured (Fig. S1b) which showed slight decrement in absorbance and Insets show the solution of CDs under Uv light. As shown in Fig. 1d, the size distribution of as synthesized CDs was verified using a zeta-seizer. The average size of the CDs was found to be 34.02 nm, which is slightly larger than the 3.5 nm obtained using TEM. It should be noted that the hydrodynamic diameter of a particle in a solvent is larger than the size of CDs measured in vacuum.

**Figure 2:**
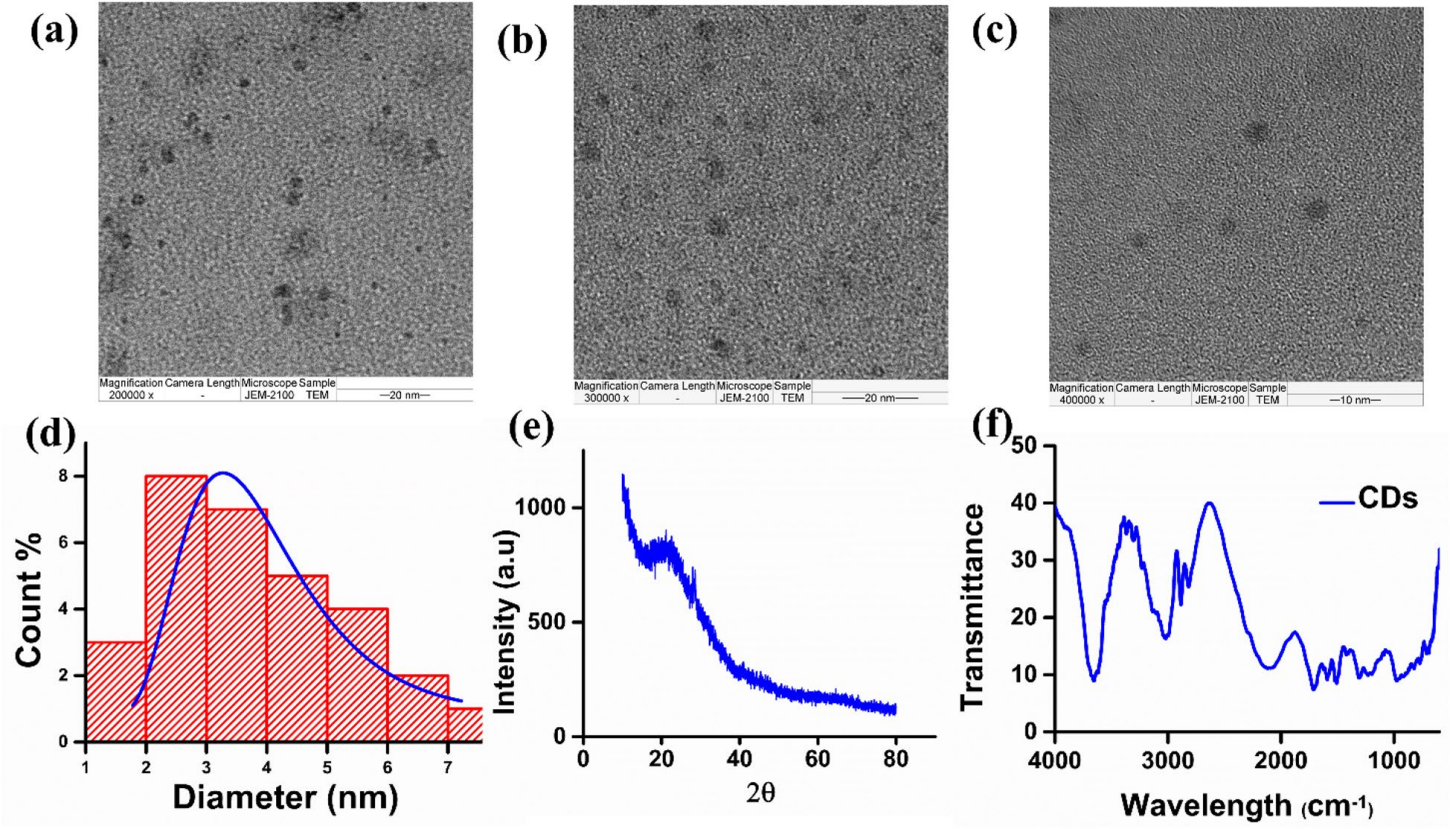
Morphology and surface analysis of CDs (**a**, **b**, **c**) HRTEM images of CDs with 200,000X, 300,000X, and 400,000X magnification. (**d**) Histogram depicting particle size distribution of CDs (**e**) X-ray diffraction pattern of CDs (**f**) FT-IR spectra of CDs.

The morphological characteristics and size of CDs were visualised using High-resolution transmission electron microscopy (HR-TEM). The TEM images captured for single CDs at multiple magnifications showed no appearance of fringes, represented in Fig. 2a, b, c. The HR-TEM image reveals a group of distinct and independent CDs with uniform black spherical spheres without aggregation, holding approximately 3.5 nm (average size), calculated using ImageJ software, and histogram^36^ in Fig. 2d calculated using Gaussian distribution in origin software^37^. A broad hump is depicted in XRD analysis pattern of CDs at 2θ = 22° (approximately), which supports the amorphous nature of CDs (Fig. 2e). Absorption bands at 3657, 3100 and 2100 cm^−1^ are imputed to the vibratory stretching of O–H, C–H and Nitrile respectively. The weak peak at 1750 and 2850 cm^−1^ corresponds to the aldehyde group (stretching vibration). The absorption bands at 1625 cm^−1^ can be ascribed to the amide group (Fig. 2f). The large negative value (− 11.0 mV) of zeta potential explains that CDs are possessed functional groups with negative charges on the surface of CDs (Fig. S8e).

### Free radical scavenging capacity

**Figure 3:**
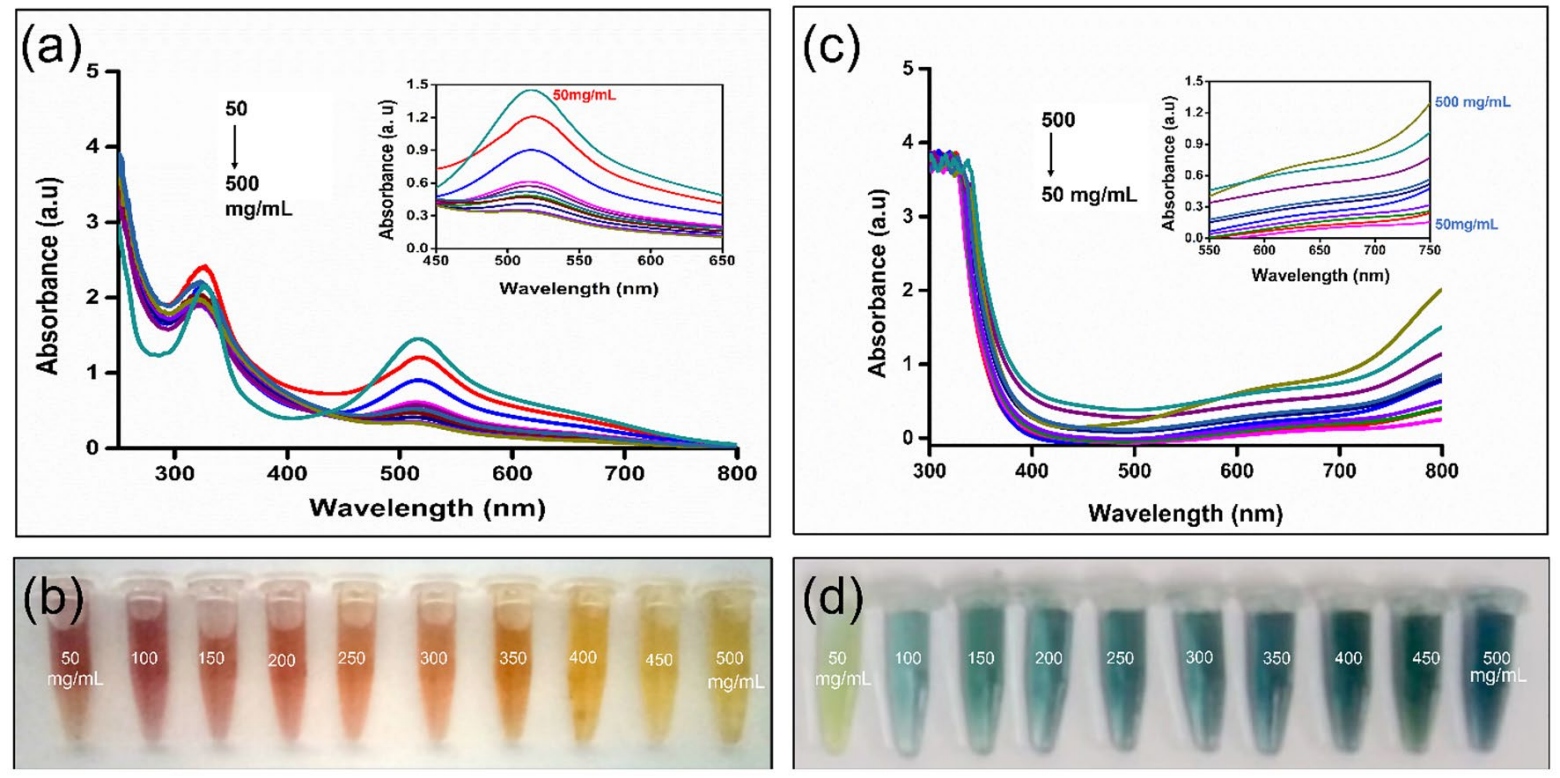
Antioxidant activity of CDs (**a**) DPPH free radical scavenging property of CDs (**b**) optical images of DPPH solution showing a gradual colour change (wine red to yellow) with the concentration increase of CDs (**c**) TAC activity by Phosphomolybdate assay (**d**) optical images of the TAC activity showing a gradual colour change (light green to deep greenish-blue) with the concentration raise of CDs.

Numerous reports have proven that CDs can be utilized as effective antioxidants^38^. Antioxidants are capable of scavenging or neutralizing free radicals. An efficient and established method to evaluate the free radical scavenging property is the DPPH assay. The presence of free radicals on nitrogen resulted for the DPPH to appear as purple color. However, upon adding DPPH to an antioxidant (CDs), the color eventually transforms to yellow due to the addition of hydrogen/radical. The ability of CDs to donate H^+^ ions is based on the presence of COOH, OH/NH_2_ surface functional groups. This results in the intensity decrease at 517 nm, observed after an incubation time of 20 min. Several volumes of CDs are added with varying concentrations (5–50 μg/mL). The absorption resembled the dose-dependent decrease, as shown as Fig. 3a. The CDs showed a gradual increase in free radical activity from 16 to 77%, with the increasing concentration accompanied by color transformation from purple to pale yellow (Fig. 3b, Table S1, Fig. S2). The half-maximal effective concentration (EC_50_) for CDs to exhibit free radical scavenging activity is 13.87 μgmL^−1^ (Fig. S3).

### Total antioxidant capacity (TAC) activity

Total antioxidant capacity is a quantitative technique that evaluates the reduction capacity of the antioxidant. The assay is based on reducing molybdenum from + VI to + V oxidation state in the presence of the antioxidant in an acidic medium. The redox reaction is indicated by the green-coloured Phosphate-Mo (V) complex formation. CDs of varying concentrations (5–50 μg/mL) were examined to determine the TAC. The reagent was added to CDs samples and incubated for 90 min. The cooled samples were measured absorbance at 695 nm. The absorbance is directly proportional to the increasing concentration (Fig. 3c). The TAC increased gradually from 108.59% to 148% as a result of increasing intensity from pale green to greenish blue (Fig. 3d, Table S2, Fig. S4). The increase is observed due to the presence of surface acids/phenolic groups on CDs. The half-maximal effective concentration (EC_50_) for CDs to exhibit total antioxidant activity is 38.72μgmL^−1^ (Fig. S5).

### Antimicrobial activity

**Figure 4:**
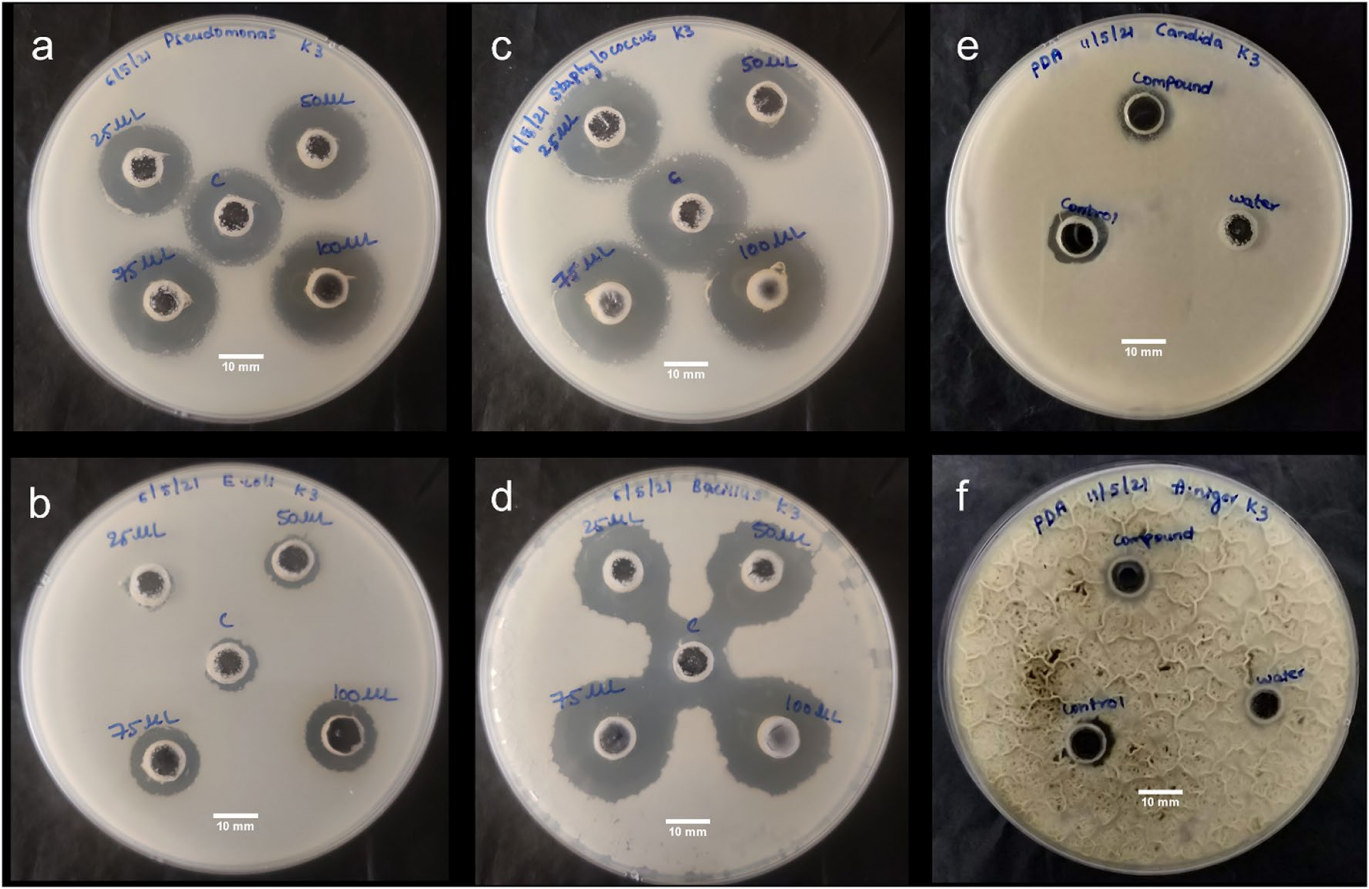
Antimicrobial activity of CDs (**a**, **b**) Antibacterial assay: Zone of inhibition against Staphylococcus aureus and Bacillus subtilis (gram-positive bacterial strains) and (**c**, **d**) Escherichia coli and pseudomonas aeruginosa (gram-negative bacterial strains), respectively (**e**, **f**) Zone of inhibition against candida and Aspergillus Niger (fungal strains) (scale bar is 10 mm for each).

The antimicrobial activity was tested by using four different bacterial strains (two gram-positive and two gram-negative) and two fungal strains (Fig. 4). The experimental results i.e. the calculated zone of inhibition for the antibacterial and antifungal activities are detailed in Table S3 and S4. The study exhibited a linear increase in the activity with increased concentration of CDs. This explains that CDs showed a dose-dependent inhibition for antibacterial and antifungal activities. The reasons behind growth inhibition can be understood as membrane lysis occurring as a result of interaction between the positive charges on the surface of CDs and negative charges on the cell membranes of the organism (bacteria and fungi)^39^. This attachment triggers physical and mechanical rupture on the bacterial/fungal membrane, allowing CDs to pass into the internal membranes. The membranes eventually collapse due to the loss of electrolytes and cytoplasm fluids. In addition, the literature survey suggests that amine groups on the surface of CDs also denature DNA^40,41^, resulting in cell apoptosis.

### Bioimaging

The CDs were further evaluated for bioimaging applications by conjugating with folic acid. The carboxylic acid group on the surface of CDs are coupled with an amino group of folic acid, enabling an amide linkage using the traditional EDC and Sulpho-NHS coupling reagents. Folic acid is water-soluble and targets specifically the folate receptors, predominantly found in cells of various tumours and cancers. The successful FA conjugation with CDs (FACDs) was evaluated by UV–Visible absorption, zeta potential estimation and fluorescence spectroscopic techniques (Fig. S6). The FACDs exhibited a shift from 270 to 260 nm in absorption peak corresponds to n-π* transitions and the peak at 350 nm was disappeared after conjugation. Further, FACDs exhibited bright blue fluorescence under exposure to the UV lamp, which means the fluorescence of CDs is not quenched upon conjugation. Also, FACDs retained excitation wavelength-dependent emission properties of CDs. The increase in zeta potential value from −11 to −0.4 mV represents effective conjugation of FA to CDs thereby decreasing the negative charges upon surface passivation. These results of FACDs render them as effective fluorescent probes in bioimaging^42^.

**Figure 5:**
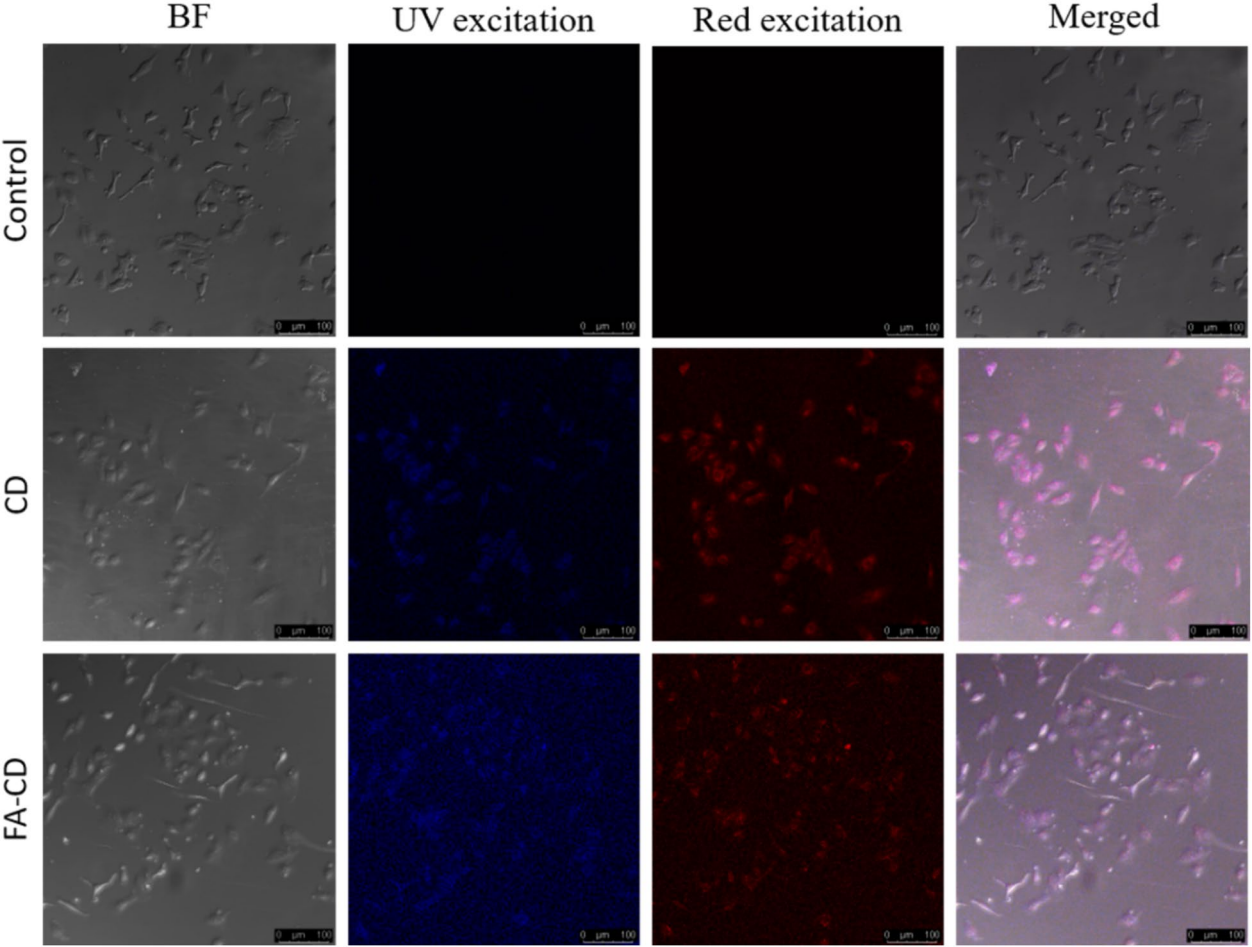
Confocal fluorescence microscopic images of MCF7 cells incubated with FACDs. (Scale bar represents 100 µm).

In order to study bioimaging, a preliminary in vitro MTT assay was performed to evaluate the biocompatibility of CDs on L929 cells (mouse fibroblast cells) (Fig. S7). The cells were treated with a varying concentration of CDs (20–1000 μg/mL) and subjected for incubation. The CDs showed good biocompatibility even at higher concentrations after 24 h of incubation. There was not much decrease in the cell viability at lower concentrations, but a slight decrease up to 80% is observed at higher concentrations. Thus, CDs exhibited low toxicity, multiple fluorescence (shown in Fig. S8) and facilitated for bioimaging studies^38,39^. To study the in vitro bio-imaging of cells, MCF7 cell lines was chosen as a folate receptor target cell and FACDs as fluorescence target probe. MCF 7 breast cancer cells were treated with CDs and FACDs. Upon 24 h of treatment, the cells were observed under the confocal microscope. A bright blue color fluorescence was observed in FACDs treated cells when compared to CDs and untreated cell lines under UV light and red-light excitation (Fig. 5). Thus, FA conjugation enables enhanced cancer cells interaction and thereby, a brighter fluorescence signal was visualised in FACDs treated cells.

## Conclusions

We have utilized a renewable green source and successfully synthesized CDs using *Azadirachta indica* leaves. The sand bath synthesis strategy utilized is cost-effective and rather simple avoiding complex and extreme conditions. The as-synthesized carbon dots demonstrated excellent excitation-dependent emission and were further observed to be biocompatible even at higher concentrations. The CDs also exhibited significant free radical scavenging property (EC_50_: 13.87 μg mL^−1^), total antioxidant activity (EC_50_:38.72 μg mL^−1^) and excellent antimicrobial activity. The synthesized CDs were modified with Folic acid on their surface and was tested for its bioimaging properties in cancer cell lines. Conjugation with FA did not affect the optical properties of CD. FACDs were observed to facilitate active targeting of cancer cells which was evident from the brighter fluorescence emission at multiple excitations.

## Supplementary Information

Supplementary Information.

## Data Availability

The datasets used and/or analyzed in this study are available in the manuscript can be asked from the corresponding author upon request.
