# Supplementary material for: Green synthesis of multi-functional carbon dots from medicinal plant leaves for antimicrobial, antioxidant, and bioimaging applications
Source: Sci Rep. 2023 Apr 19;13:6371. doi: 10.1038/s41598-023-33652-8 (PMC10115846; doi:10.1038/s41598-023-33652-8)
Supplement: Supplementary file 1 — Supplementary Information. [file 41598_2023_33652_MOESM1_ESM.docx]

Green Synthesis of Multi-Functional Carbon Dots from Medicinal Plant leaves for Anti-microbial, Antioxidant, and Bioimaging applications

Gangaraju Gedda^a^, Sri Amruthaa Sankaranarayanan^b^, Chandra Lekha Putta^b^, Krishna Kanthi Gudimella^c^*, Aravind Kumar Rengan^b^*, Wubshet Mekonnen Girma^d*^

^a^Department of Chemistry, School of Engineering, Presidency University, Bangalore,560064, Karnataka, India

^b^Department of Biomedical Engineering, Indian Institute of Technology Hyderabad, Sangareddy, Telangana-502285, India

^c^Department of Chemistry, School of Science, GITAM (Deemed to be University), Rudraram, Telangana-502329, India

^d^Department of Chemistry, College of Natural Science, Wollo University, P.O. Box:1145, Dessie, Ethiopia

*Corresponding authors: Krishna Kanthi Gudimella, Aravind Kumar Rengan and Wubshet Mekonnen Girma

Department of Chemistry, College of Natural Science, Wollo University, P.O. Box:1145, Dessie, Ethiopia

E-mail: wubshet.mekonnen@wu.edu.et

Tel.: +251-0-910804026

Table S1: Carbon dots derived from medicinally important precursors and applications.

| Precursor | Method | Fluorescence colour | Application | Reference |
| --- | --- | --- | --- | --- |
| Neem gum + ethanol | Chemical oxidation | Green | NA | ^1^ |
| Azadirachta indica Leaves | Hydrothermal | Blue | Detection of H_2_O_2_ and Ascorbic Acid | ^2^ |
| Pea seasame | Hydrothermal | Blue | Bioimaging,labelling | ^3^ |
| Tulsi leaves | hydrothermal | blue | Bioimaging Patterning agents | ^4^ |
| bamboo leaves + Branched polyethylenimine | Hydrothermal | blue | copper(II) ion detection | ^5^ |
| Mustard seeds | hydrothermal | blue | peroxidase-like activity for colorimetric detection of H_2_O_2_ and ascorbic acid | ^6^ |
| gardenia fruit | hydrothermal | blue | Detection of Hg^2+^ and cysteine | ^7^ |
| Pinellia Ternata +ethylenediamine | hydrothermal | blue | Determination of chromium (VI) | ^8^ |
| pomelo | hydrothermal | blue | Tetracycline sensing | ^9^ |
| Rose flowers +P_2_O_5_ | microwave | blue | Tetracycline sensing | ^10^ |
| Ginko leaves | hydrothermal | blue | Detection of salazosulfapyridine | ^11^ |
| Aurantii fructus immaturus | High-temperature pyrolysis | NA | Antihyperuricemic and anti-gouty arthritis activities | ^12^ |
| Honey | hydrothermal | blue | Bioimaging | ^13^ |
| Fenugreek seeds | hydrothermal | Blue-green | Lighting Applications | ^14^ |
| Orange peels + EDTA | hydrothermal | Blue | Cr (VI) and ascorbic acid detection | ^15^ |
| Neem leaves | Sandbath assisted method | Blue | Antioxidant activity, anti-bacterial, antifungal,  bioimging | This work |

Figure S1 : Free radical scavenging activity


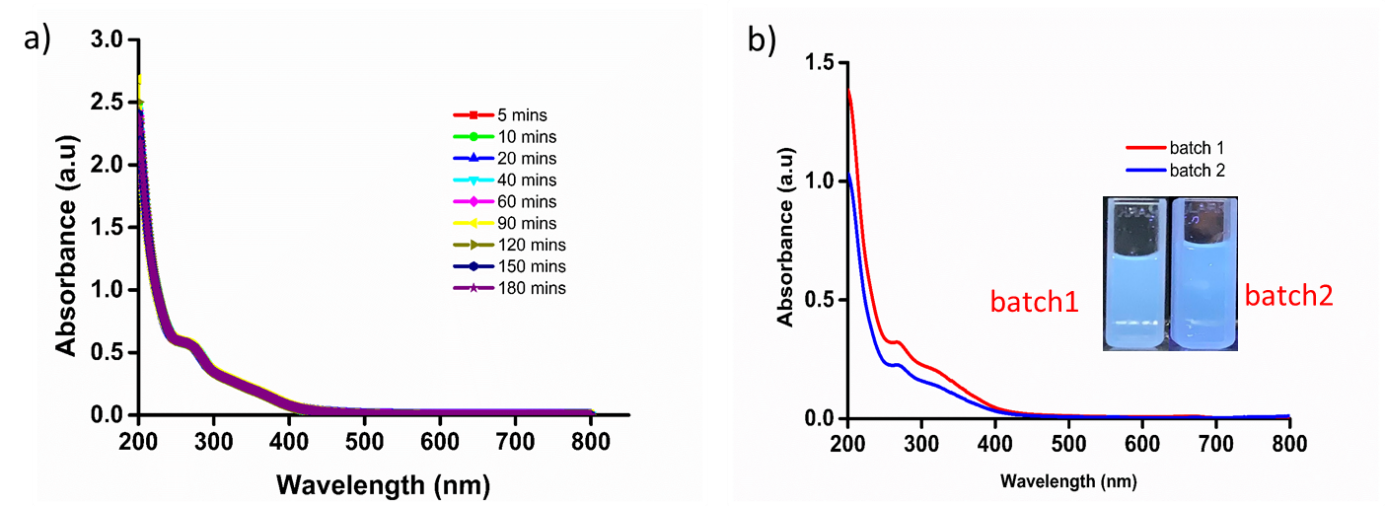


Figure S1: Stability studies of CDs a) UV- Visible spectra of CDs placed under UV light for a time period of 5-180 min b) UV- Visible spectra of CDs measured before and after two months and insets are images of CDs under UV light.





Figure S2: DLS -deconvolution study of CDs


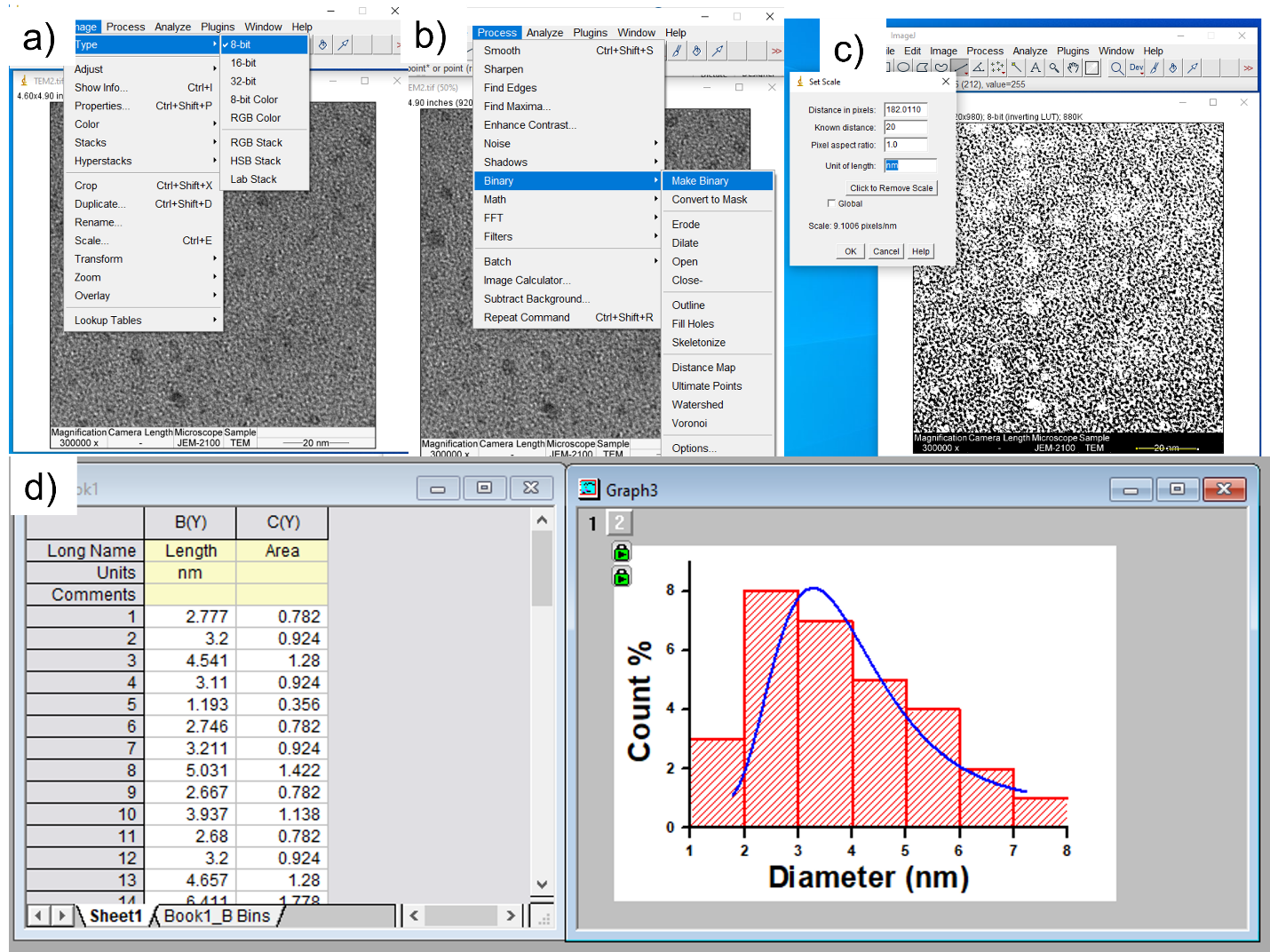


Figure S3:Size calculation of CDs in TEM image using ImageJ software ( a,b,c) Sequential processing of TEM image to determine particle size using Image J software d) histogram showing average particle size of CDs using origin software.





Figure S4: Free radical scavenging activity of CDs and ascorbic acid(reference).

Table S2: Free radical scavenging activity of CDs and Ascorbic acid (standard reference)

| **Concentration (μg/μL)** | **Free radical scavenging activity(%)** | |
| --- | --- | --- |
|  | **CDs** | **Ascorbic acid** |
| 5 | 16.47±0.72 | 27.7687 |
| 10 | 37.57±1.57 | 47.0892 |
| 15 | 49.71±2.28 | 61.3298 |
| 20 | 58.34±1.51 | 69.6731 |
| 25 | 64.01±2.60 | 73.1786 |
| 30 | 66.57±3.12 | 81.0923 |
| 35 | 69.75±2.27 | 84.087 |
| 40 | 72.29±1.14 | 86.5467 |
| 45 | 75.98±2.79 | 91.7964 |
| 50 | 77.09±1.85 | 93.5072 |





Figure S5: Half maximal effective concentration (EC_50_) of Free radical scavenging activity.





Figure S6: Total Antioxidant Activity CDs and Ascorbic acid (standard reference)

Table S3: Total Antioxidant Activity CDs

| **Concentration (μg/μL)** | **Total Antioxidant Activity** | |
| --- | --- | --- |
|  | CDs | Ascorbic acid |
| 5 | 108.59±2.42 | 118.67 |
| 10 | 111.21±4.56 | 130.4 |
| 15 | 113.34±5.66 | 141.01 |
| 20 | 116.87±1.84 | 148.26 |
| 25 | 121.03±1.05 | 153.68 |
| 30 | 123.29±4.16 | 162.22 |
| 35 | 132.44±3.62 | 170.22 |
| 40 | 141.62±2.081 | 173.75 |
| 45 | 141.62±3.08 | 177.81 |
| 50 | 148.18±1.40 | 184.59 |
|  |  |  |





Figure S7: Half maximal effective concentration (EC_50_) of total antioxidant activity

Table S4: Antibacterial activity of CDs

| Volume sμL | Name of bacterial strains  Zone of inhibition, mm n=3 | | | |  |
| --- | --- | --- | --- | --- | --- |
|  |  |  |  |  |  |
|  | *Bacillus  Subtilis* | *Staphylococcus  Aureus* | *E.Coli* | *Pseudomonas  Aeruginosa* |  |
| 25 | 20.001 | 22.078 | 9.808 | 18.822 |  |
| 50 | 22.242 | 22.303 | 12.369 | 20.321 |  |
| 75 | 26.022 | 24.666 | 16.211 | 20.828 |  |
| 100 | 25.413 | 24.622 | 15.44 | 24 |  |
| control | 19.204 | 24.614 | 11.152 | 20.746 |  |

Table S5: Antifungal activity of CDs

| Name of the fungal  Strain | Name of the Sample  Zone of inhibition, mm (n=3) | | |
| --- | --- | --- | --- |
|  | Compound | Water | Control |
| *Candida Albicans* | 13.22±0.13   \|  \| \| --- \| \|  \| | -- | 12.806±0.76 |
| *Aspergillus Niger* | 9.527±0.29 | -- | 9.93±0.07 |


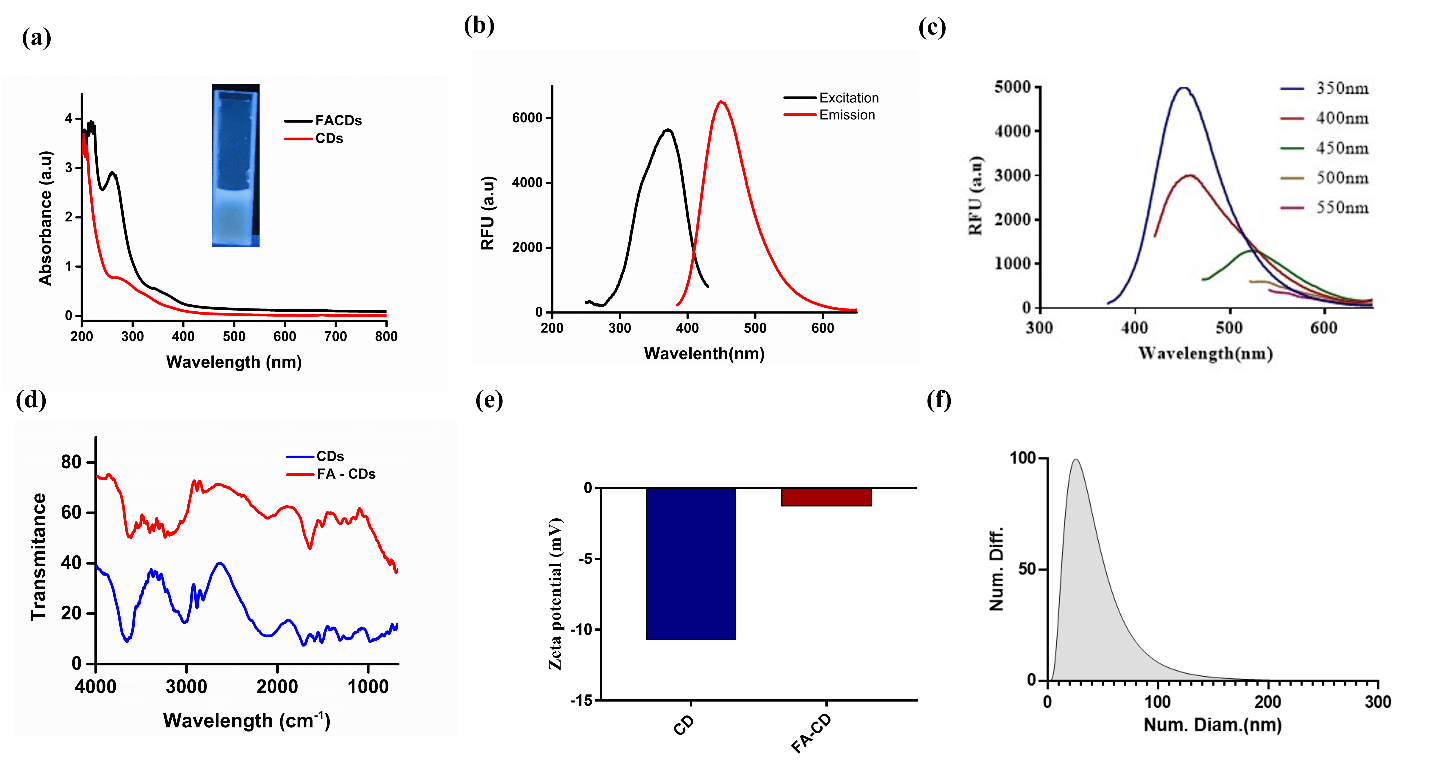


Figure S8: Optical characterizations of FA CDs using (a)UV–Visible absorption, (b)Excitation -emission spectra (c)Fluorescence spectra (d) FTIR spectra (e) zeta potential, and (f) DLS analysis.


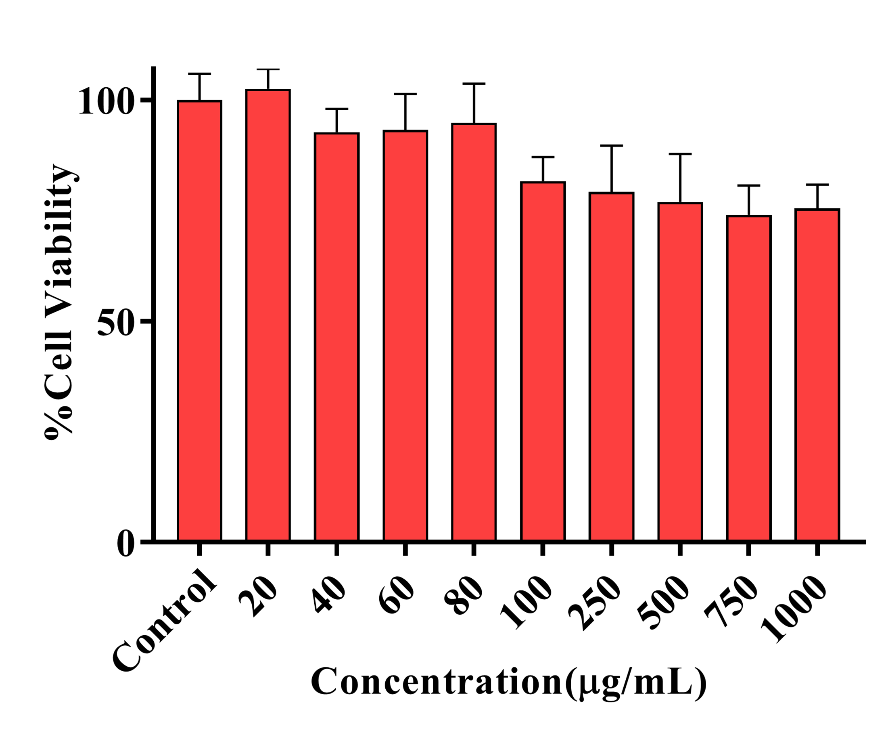


Figure S9: Biocompatibility of FACDs


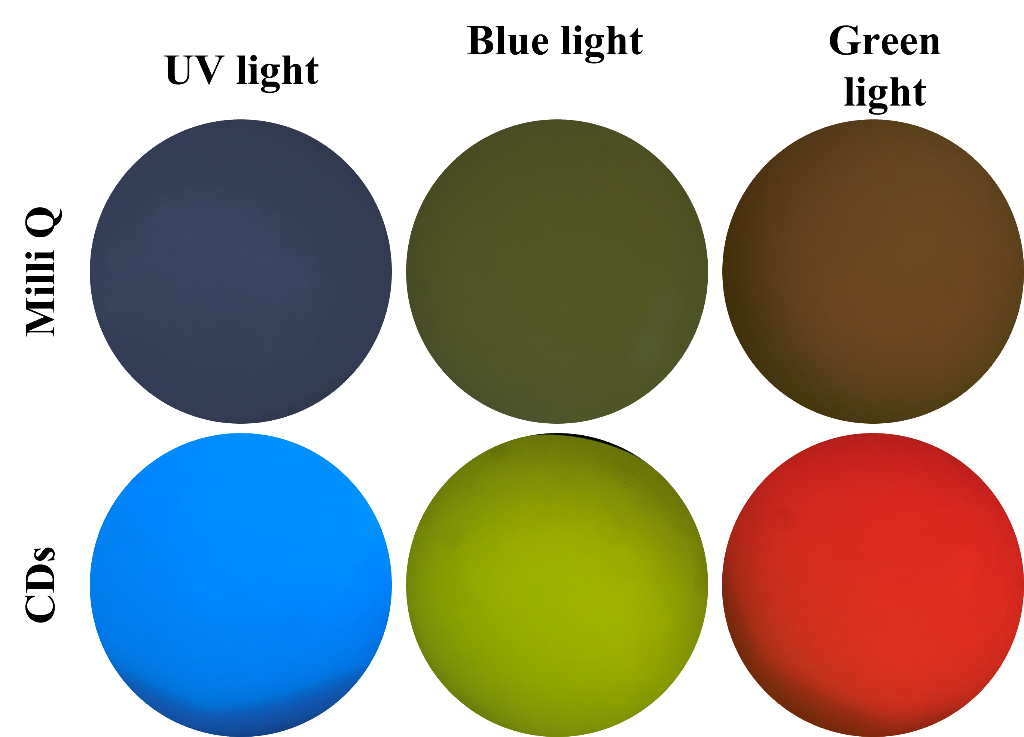


Figure S10: Multi colour fluorescence of CDs

**References**

1. Phadke, C., Mewada, A., Dharmatti, R., Thakur, M., Pandey, S. & Sharon, M. (2015). Biogenic Synthesis of Fluorescent Carbon Dots at Ambient Temperature Using Azadirachta indica (Neem) gum. *J Fluoresc* **25**, 1103-7.

2. Yadav, P. K., Singh, V. K., Chandra, S., Bano, D., Kumar, V., Talat, M. & Hasan, S. H. (2019). Green Synthesis of Fluorescent Carbon Quantum Dots from Azadirachta indica Leaves and Their Peroxidase-Mimetic Activity for the Detection of H(2)O(2) and Ascorbic Acid in Common Fresh Fruits. **5**, 623-632.

3. Su, Q., Gan, L., Liu, J. & Yang, X. (2020). Carbon dots derived from pea for specifically binding with Cryptococcus neoformans. *Analytical Biochemistry* **589**, 113476.

4. Bhatt, S., Bhatt, M., Kumar, A., Vyas, G., Gajaria, T. & Paul, P. (2018). Green route for synthesis of multifunctional fluorescent carbon dots from Tulsi leaves and its application as Cr(VI) sensors, bio-imaging and patterning agents. *Colloids and Surfaces B: Biointerfaces* **167**, 126-133.

5. Liu, Y., Zhao, Y. & Zhang, Y. (2014). One-step green synthesized fluorescent carbon nanodots from bamboo leaves for copper(II) ion detection. *Sensors and Actuators B: Chemical* **196**, 647-652.

6. Chandra, S., Singh, V. K., Yadav, P. K., Bano, D., Kumar, V., Pandey, V. K., Talat, M. & Hasan, S. H. (2019). Mustard seeds derived fluorescent carbon quantum dots and their peroxidase-like activity for colorimetric detection of H2O2 and ascorbic acid in a real sample. *Analytica Chimica Acta* **1054**, 145-156.

7. Sun, D., Liu, T., Wang, C., Yang, L., Yang, S. & Zhuo, K. (2020). Hydrothermal synthesis of fluorescent carbon dots from gardenia fruit for sensitive on-off-on detection of Hg2+ and cysteine. *Spectrochimica Acta Part A: Molecular and Biomolecular Spectroscopy* **240**, 118598.

8. Dai, J. & Wang, Y. (2019). Nitrogen-doped carbon quantum dots with Pinellia ternataas carbon source for high sensitive determination of chromium (VI). *Applied Ecology and Environmental Research* **17**, 12139-12153.

9. Qi, H., Huang, D., Jing, J., Ran, M., Jing, T., Zhao, M., Zhang, C., Sun, X., Sami, R. & Benajiba, N. (2022). Transforming waste into value: pomelo-peel-based nitrogen-doped carbon dots for the highly selective detection of tetracycline. *RSC Advances* **12**, 7574-7583.

10. Feng, Y., Zhong, D., Miao, H. & Yang, X. (2015). Carbon dots derived from rose flowers for tetracycline sensing. *Talanta* **140**, 128-133.

11. Jiang, X., Qin, D., Mo, G., Feng, J., Yu, C., Mo, W. & Deng, B. (2019). Ginkgo leaf-based synthesis of nitrogen-doped carbon quantum dots for highly sensitive detection of salazosulfapyridine in mouse plasma. *Journal of Pharmaceutical and Biomedical Analysis* **164**, 514-519.

12. Wang, S., Zhang, Y., Kong, H., Zhang, M., Cheng, J., Wang, X., Lu, F., Qu, H. & Zhao, Y. (2019). Antihyperuricemic and anti-gouty arthritis activities of Aurantii fructus immaturus carbonisata-derived carbon dots. *Nanomedicine* **14**, 2925-2939.

13. Yang, X., Zhuo, Y., Zhu, S., Luo, Y., Feng, Y. & Dou, Y. (2014). Novel and green synthesis of high-fluorescent carbon dots originated from honey for sensing and imaging. *Biosensors and Bioelectronics* **60**, 292-298.

14. Urushihara, N., Hirai, T., Dager, A., Nakamura, Y., Nishi, Y., Inoue, K., Suzuki, R., Tanimura, M., Shinozaki, K. & Tachibana, M. (2021). Blue–Green Electroluminescent Carbon Dots Derived from Fenugreek Seeds for Display and Lighting Applications. *ACS Applied Nano Materials* **4**, 12472-12480.

15. Wang, M., Shi, R., Gao, M., Zhang, K., Deng, L., Fu, Q., Wang, L. & Gao, D. (2020). Sensitivity fluorescent switching sensor for Cr (VI) and ascorbic acid detection based on orange peels-derived carbon dots modified with EDTA. *Food Chemistry* **318**, 126506.
